# Supplementary material for: An open-source framework for large-scale, flexible evaluation of biomedical text mining systems
Source: J Biomed Discov Collab. 2008 Jan 29;3:1. doi: 10.1186/1747-5333-3-1 (PMC2276192; doi:10.1186/1747-5333-3-1)
Supplement: Additional file 1 — Evaluation platform source code. The additional file contains the software evaluation framework discussed in this paper. The most current release can be downloaded from SourceForge.net [1]. See the accompanying README file for instructions on installation and use. Also included in the distribution is a collection of UIMA wrappers for some commonly used BioNLP tools and annotated corpora. [file 1747-5333-3-1-S1.zip › bionlp_uima_repository-1.0.1/docs/api/src-html/edu/uchsc/ccp/uima/cc/summary/comparison/AnnotationGroup.html]

```
001    package edu.uchsc.ccp.uima.cc.summary.comparison;
002    
003    import java.io.PrintStream;
004    import java.util.ArrayList;
005    import java.util.List;
006    
007    public class AnnotationGroup {
008        private AnnotationGroupID groupID;
009    
010        private Integer annotationSetID;
011    
012        private Integer annotatorID;
013    
014        private List<String> annotationTypeList;
015    
016        public AnnotationGroup() {
017            this.groupID = null;
018            this.annotationSetID = null;
019            this.annotatorID = null;
020            this.annotationTypeList = new ArrayList<String>();
021        }
022    
023        /* returns true if all fields in the AnnotationGroup are non-null */
024        public boolean isValid() {
025            if (groupID != null & annotationSetID != null & annotatorID != null & annotationTypeList.size() != 0) {
026                return true;
027            } else {
028                return false;
029            }
030        }
031    
032        public Integer getAnnotationSetID() {
033            return annotationSetID;
034        }
035    
036        public void setAnnotationSetID(Integer annotationSetID) {
037            this.annotationSetID = annotationSetID;
038        }
039    
040        public List<String> getAnnotationTypeList() {
041            return annotationTypeList;
042        }
043    
044        public void setAnnotationTypeList(List<String> annotationTypeList) {
045            this.annotationTypeList = annotationTypeList;
046        }
047    
048        public void addAnnotationType(String type) {
049            this.annotationTypeList.add(type);
050        }
051    
052        public Integer getAnnotatorID() {
053            return annotatorID;
054        }
055    
056        public void setAnnotatorID(Integer annotatorID) {
057            this.annotatorID = annotatorID;
058        }
059    
060        public AnnotationGroupID getGroupID() {
061            return groupID;
062        }
063    
064        public void setGroupID(AnnotationGroupID groupID) {
065            this.groupID = groupID;
066        }
067    
068        
069        public void print(PrintStream ps) {
070            String typeStr = "";
071            for (String type : this.annotationTypeList) {
072                typeStr += (type + ", ");
073            }
074            typeStr = typeStr.substring(0, typeStr.lastIndexOf(","));
075            ps.println("Annotation Group: ID: " + this.groupID + " Annotator: " + this.getAnnotatorID() + " AnnotationSet: " + this.getAnnotationSetID() + " Type(s): " + typeStr);
076        }
077        
078    }
```
